# Percutaneous sclerotherapy with OK-432 for lymphocele after pelvic or para-aortic lymphadenectomy: preliminary results

**DOI:** 10.1186/s42155-022-00332-z

**Published:** 2022-10-20

**Authors:** Eiji Kashiwagi, Yusuke Ono, Hiroki Yano, Shinya Kosai, Keisuke Nagai, Kaishu Tanaka, Hiroki Higashihara, Noriyuki Tomiyama

**Affiliations:** Department of Diagnostic and Interventional Radiology, Osaka University Graduate School of Medicine, 2-2 Yamadaoka, Suita, Osaka, Japan

**Keywords:** OK-432, Sclerotherapy, Lymphocele

## Abstract

**Background:**

Lymphoceles can result from disruption of lymphatic vessels after surgical procedures in areas with extensive lymphatic networks. Percutaneous catheter drainage with sclerotherapy can be performed for the treatment of lymphoceles. OK-432 has been used to treat benign cysts, such as lymphangioma and ranula. Therefore, we aimed to report the efficacy and safety of sclerotherapy using OK-432 for postoperative lymphoceles. This study retrospectively analyzed 16 patients who underwent sclerotherapy using OK-432 for postoperative pelvic and para-aortic lymphoceles between April 1, 2012, and March 31, 2020. All the patients underwent percutaneous drainage before sclerotherapy. The indications for sclerotherapy were persistent drainage tube output of greater than 50 mL per day and recurrent lymphoceles after percutaneous drainage. If less than 20 mL per day was drained after sclerotherapy, the tube was removed. When the drainage tube output did not decrease to less than 20 mL per day after the first sclerotherapy, the second sclerotherapy was performed 1 week later. Technical success was defined as the completion of drainage and sclerotherapy procedures. Clinical success was defined as the resolution of the patient’s symptoms resulting from lymphoceles without surgical intervention. This study also evaluated the complications of sclerotherapy and their progress after sclerotherapy.

**Results:**

The mean initial lymphocele size and drainage duration after sclerotherapy were 616 mL and 7.1 days, respectively. The technical success rate and clinical success rate were 100% and 93%, respectively. Thirteen patients were treated by one-session sclerotherapy and three patients were treated by two-session sclerotherapy. Minor complications (fever) were observed in eight patients (50%). A major complication (small bowel fistula) was observed in one patient (7%). No recurrence of lymphoceles was observed during the mean follow-up period of 17 months.

**Conclusion:**

Sclerotherapy with OK-432 is an effective therapeutic method for postoperative lymphoceles. Although most complications are minor, a small bowel fistula was observed in one patient.

## Background

Lymphoceles are postoperative cystic collections of lymph fluid surrounded by a fibrous wall lacking epithelium. They can result from disruption of lymphatic vessels after surgical procedures in areas with extensive lymphatic networks. Lymphoceles can cause hydronephrosis, infection, abdominal pain, leg edema, and deep venous thrombosis (Karcaaltincaba & Akhan, 2005a; vanSonnenberg et al., 1986). The incidence rate of symptomatic lymphoceles ranges from 2% to 9%, depending on the type of surgery (Goßler et al., 2021; Heers et al., 2015; Zikan et al., 2015). Asymptomatic lymphoceles resolve spontaneously without treatment (Dodd et al., 1970); however, symptomatic lymphoceles might require treatment. Treatment options for symptomatic lymphoceles include percutaneous fine-needle aspiration, percutaneous catheter drainage, percutaneous catheter drainage with sclerotherapy, lymphatic embolization, and surgery (Ten Hove et al., 2021). Minimally invasive treatment has become the first treatment option (Ten Hove et al., 2021). Percutaneous fine-needle aspiration is no longer performed due to its high recurrence rate, the need for frequent punctures, and infection risk (Jensen et al., 1986; Karcaaltincaba & Akhan, 2005b). Lymphatic embolization has been reported as a treatment option because of its high success and low recurrence rates. However, lymphatic embolization is often selected when the patient does not respond to previous treatment (e.g., percutaneous catheter drainage with or without sclerotherapy), and this procedure requires a learning curve (Ten Hove et al., 2021; Addo et al., 2018; Baek et al., 2016). In the past, external drainage and internal marsupialization via laparotomy or laparoscopy were the treatment of choice. However, surgery has a relatively high risk of complications (e.g., injury to the bladder, ureter, and ileus) and long hospitalization (Ten Hove et al., 2021; Gill et al., 1995). Currently, percutaneous catheter drainage with or without sclerotherapy is often chosen for symptomatic lymphoceles and has achieved a high success rate (Ten Hove et al., 2021). Various agents have been used for sclerotherapy, including povidone-iodine, ethanol, tetracycline, bleomycin, and fibrin glue (Alago et al., 2013; Akhan et al., 2007; Chin et al., 2007; Filippiadis et al., 2017). However, a consensus regarding effective sclerosing agents for lymphoceles has not yet been reached. OK-432 (Picibanil^Ⓡ^, Chugai Pharmaceutical Co., Ltd., Tokyo, Japan) is a lyophilized mixture of a low-pathogenic strain of *Streptococcus pyogenes* (Su) incubated with benzylpenicillin. It is widely used to treat malignant pleural effusions, chylothorax, and pneumothorax (Kasahara et al., 2006; Ono et al., 2010; Takeda et al., 2006). OK-432 sclerotherapy has been reported to be effective in the treatment of benign cysts, such as lymphangioma and ranula (Ogita et al., 1994; Roh, 2006). The mechanism of action of OK-432 on benign cysts is to immediately induce inflammation, causing inflammatory cells to invade the cyst and cause the cyst to adhere (Fujino et al., 2003). Although sclerotherapy with OK-432 for postoperative lymphoceles in the inguinal or axillary region has been reported (Uyulmaz et al., 2020), there have been no reports of sclerotherapy with OK- 432 for lymphoceles after pelvic and para-aortic lymphadenectomy in English literature. This study aimed to report the efficacy and safety of sclerotherapy of lymphoceles using OK-432.

## Material and Methods

### Patients

This study was approved by the Institutional Review Board of Osaka University Hospital, Japan. We reviewed the electronic medical records of all patients who underwent percutaneous drainage of postoperative pelvic and para-aortic lymphoceles between April 1, 2012, and March 31, 2020, at our institution. A total of 50 lymphoceles in 45 patients (5 male, 40 female) were treated by percutaneous drainage. There were 45 patients, 26 patients who were completely treated with percutaneous drainage alone and 3 patients who were completely treated with sclerotherapy using minocycline alone were excluded from the study. Finally, 16 patients who underwent sclerotherapy with OK-432 were included in this retrospective study. Sixteen patients were diagnosed with 16 lymphoceles. Fifteen patients underwent surgery for gynecological cancer, and one patient underwent surgery for rectal cancer. Patient characteristics are shown in Table 1. The diagnosis of lymphocele was based on clinical course and imaging findings. Lymphocele infection was confirmed by fever, elevated white blood cell count and C-reactive protein level, or bacterial culture of the drainage fluid. None of the patients had previously received surgical treatment for lymphoceles, but one patient was treated with OK-432 after failing to respond to sclerotherapy with ethanol, povidone-iodine, fibrin glue, and minocycline.

**Table 1:** Characteristics of the patients

	Total cases (*n* = 16)
Mean age, years	56 (34ｰ71)
Sex	
Male	0
Female	16
Cancer types	
Gynecological cancer	15
Rectal cancer	1
Site of lesion	
Pelvic	14
Para-aortic	2
Primary symptoms	
Fever	4
Abdominal pain	5
Hydronephrosis	6
Lower extremity edema	1
Infection of lymphoceles	8
Previous treatment^a^	1

^a^One patient was treated with ethanol, povidone-iodine, fibrin glue, and minocycline before sclerotherapy with OK-432

### Procedures

**Fig. 1:**
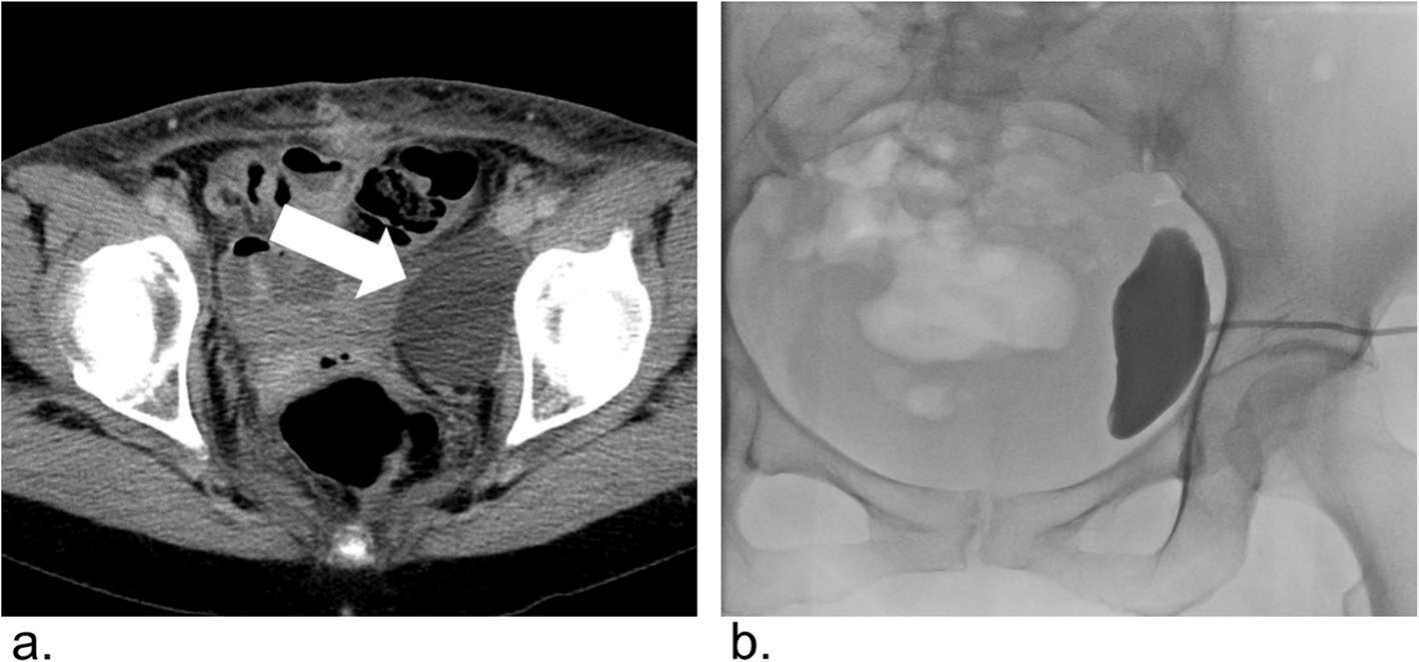
**a** Contrast-enhanced computed tomography (CT) after gynecological cancer surgery in a 60-year-old woman presenting with left low leg lymphedema. CT shows left pelvic lymphocele (white arrow). **b** Cavitogram shows no leakage of the lymphocele

All the patients underwent percutaneous drainage before sclerotherapy. Two patients underwent repeat drain placement due to recurrent lymphoceles after initial drain removal. Percutaneous drainage was performed under ultrasound or computed tomography (CT) guidance by an experienced interventional radiologist. A 7- to 8.5-French pigtail catheter (Dawson-Mueller Drainage Catheters, Cook Medical) was placed into the cavity. If there was residual fluid, the drainage tube was exchanged as needed to ensure sufficient drainage. Fifteen patients underwent sclerotherapy because of persistent drainage tube output of greater than 50 mL/day or recurrent lymphoceles after percutaneous drainage. One patient underwent initial percutaneous drainage and consecutive sclerotherapy in one session, based on the operator’s judgment. Klinische Einheit (KE) is used to express the preparation dosage. One KE of OK-432 contains 0.1 mg of dried cocci. OK-432 solution was prepared by dissolving one KE of OK-432 in 10 mL of half diluted contrast media (Urografin-60; Bayer, Leverkusen, Germany) with saline. After emptying the cavity, a cavitogram was obtained to exclude leakage from the lymphoceles (Fig. 1). The OK-432 solution was injected into the cavity and left there for 2 hours, after which catheter was allowed to drain. The volume of OK-432 used for sclerotherapy was determined based on estimated volume of the residual cavity by a cavitogram immediately before sclerotherapy. If the drainage tube drained less than 20 mL per day after sclerotherapy, the tube was removed. When the drainage tube output did not decrease to less than 20 mL per day on the sixth day after the first sclerotherapy, the second OK-432 sclerotherapy were performed on the seventh day. All procedures were performed under local anesthesia during hospitalization. After discharge, the patients were observed by clinical follow-up and abdominal CT. Technical success was defined as the completion of drainage and sclerotherapy procedures resulting in adequate decrease of the drainage tube output and tube removal. Clinical success was defined as the resolution of the patient’s symptoms resulting from lymphoceles without surgical intervention. The estimated lymphocele volume was calculated by CT volumetry using a software Aquarius iNtuition Edition version 4.4.13^Ⓡ^. Complications were evaluated according to the classification of the CIRSE classification system (Filippiadis et al., 2017). Thus, Major complications were defined as grade 2 to grade 6. Minor complications were defined as grade 1.

### Statistical analysis

Data analyses were performed with EZR (Saitama Medical Center, Jichi Medical University, Saitama, Japan), which is a graphical user interface for R (The R Foundation for Statistical Computing, Vienna, Austria). The results are presented as mean (standard deviation [SD]) for quantitative variables and frequency (percentage) for qualitative variables. In the statistical analysis, Pearson’s correlation coefficient was used to assess the correlation between the initial lymphocele size and drainage duration after sclerotherapy. Statistical significance was considered at *p*-value of < 0.05.

## Results

**Fig. 2:**
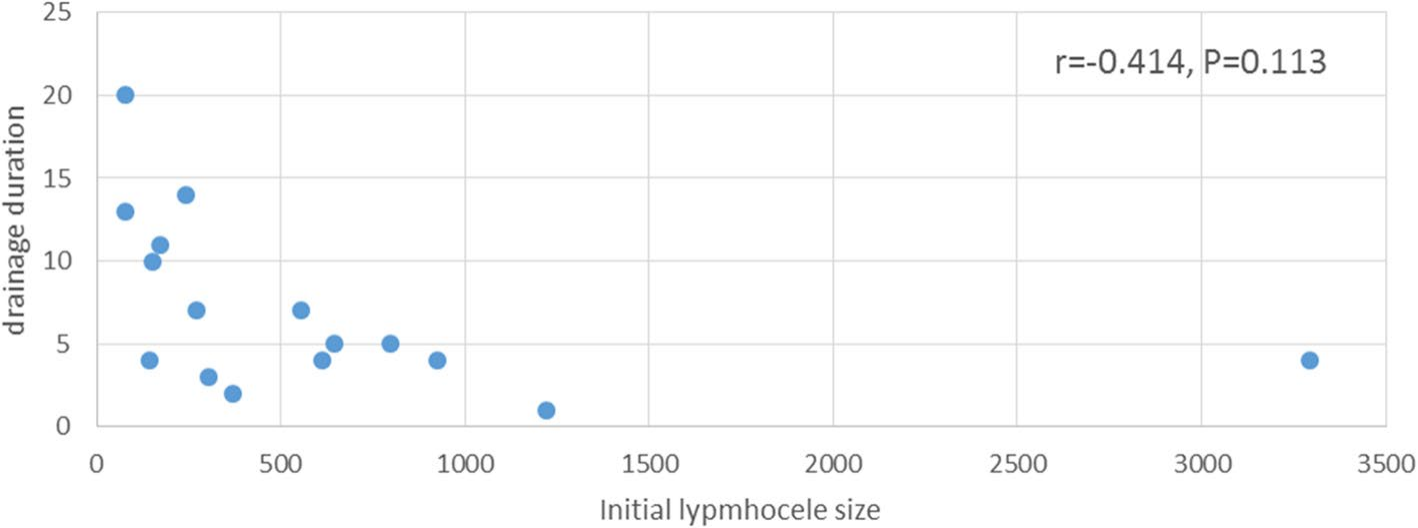
Correlations between initial lymphocele size and drainage duration

**Fig. 3:**
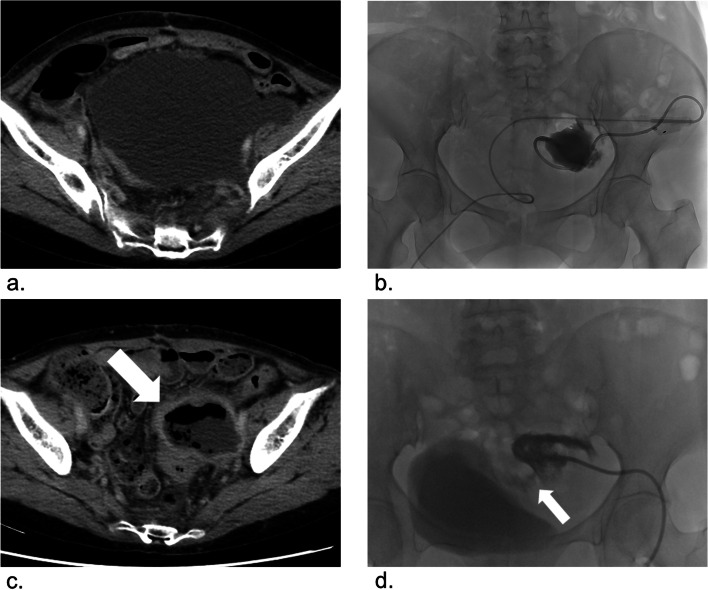
**a** Contrast-enhanced computed tomography (CT) after gynecological cancer surgery in a 69-year-old woman presenting with abdominal pain. CT shows pelvic lymphocele. **b** Cavitogram shows lymphocele reduction, sclerotherapy with OK-432 was performed. **c** One month after sclerotherapy, CT showed abscess in the pelvic (white arrow). **d** Cavitogram shows a small bowel fistula (white arrow)

The mean initial lymphocele size was 616 (range, 76–3295) mL. The mean drainage duration after sclerotherapy was 7.1 (Karcaaltincaba & Akhan, 2005a; vanSonnenberg et al., 1986; Goßler et al., 2021; Heers et al., 2015; Zikan et al., 2015; Dodd et al., 1970; Ten Hove et al., 2021; Jensen et al., 1986; Karcaaltincaba & Akhan, 2005b; Addo et al., 2018; Baek et al., 2016; Gill et al., 1995; Alago et al., 2013; Akhan et al., 2007; Chin et al., 2007; Filippiadis et al., 2017; Sacks et al., 2003; Kasahara et al., 2006; Ono et al., 2010; Takeda et al., 2006) days. Pearson’s correlation coefficient showed moderately negative correlation (*r* = − 0.414) between the initial lymphocele size and drainage duration after sclerotherapy, but the association was not statistically significant (*p* = 0.113) (Fig. 2). The mean injection volume of OK-432 was 1.9 KE. The technical success rate was 100% (16/16). The clinical success rate was 93% (15/16). One patient with hydronephrosis did not improve. Three patients were treated by two-session sclerotherapy (Table 2). The patients’ lymphoceles were not infected and the drainage tube output tended to be high volume the day before the first sclerotherapy (Table 3). Minor complications (grade 1) were observed in eight patients (50%), who presented with fever. Major complication (grade 3) was observed in one patient (7%). One month after the sclerotherapy, the patient presented with fever. CT revealed an abscess in the pelvis. Percutaneous drainage was performed, and a cavitogram showed a small bowel fistula (Fig. 3). Consequently, the small bowel fistula was resolved only by percutaneous drainage. No recurrence of lymphoceles was observed during the mean follow-up period of 17 (range, 2.5–49.8) months.

**Table 2:** Results of percutaneous sclerotherapy

	Total cases (*n* = 16)
Mean initial lymphocele size, mL	616 (76–3295)
Mean drainage duration after sclerotherapy, days	7.1 (1–20)
Mean volume of OK-432, KE^a^	1.9 (1–4)
Number of sessions	
One	13
Two	3
Technical success, n (%)	16 (100%)
Clinical success, n (%)	15 (93%)
Complication	
Major	1
Minor	8

^a^The Klinische Einheit (KE) is used to express the dosage of preparation; 1 KE of OK-432 contains 0.1 mg of dried cocci

**Table 3:** Cases of sclerotherapy

Case	Number of sessions	Volume of OK-432	Drainage output (mL/24 h)^a^	Infection
1^b^	2	2KE+4KE	50	–
2	2	2KE+2KE	360	–
3	1	2KE	100	–
4	1	2KE	130	–
5	1	2KE	200	Yes
6	1	2KE	120	Yes
7	1	2KE	100	Yes
8^c^	1	2KE	–	–
9	1	2KE	100	Yes
10	1	1KE	280	–
11^d^	1	1KE	30	Yes
12^d^	1	1KE	25	Yes
13	1	2KE	70	Yes
14	1	1KE	100	Yes
15	2	2KE+2KE	810	–
16	1	2KE	60	Yes

^a^Drainage tube output the day before the first sclerotherapy

^b^The case was treated with OK-432 after failing to respond to sclerotherapy with other sclerosing agents

^c^The case underwent initial percutaneous drainage and consecutive sclerotherapy in one session

^d^The cases were treated for recurrent lymphoceles after percutaneous drainage

## Discussion

A variety of sclerosing agents have been used for sclerotherapy of lymphoceles in previous reports (Alago et al., 2013; Akhan et al., 2007; Chin et al., 2007; Filippiadis et al., 2017). This study is the first reports of sclerotherapy with OK- 432 for lymphoceles after pelvic and para-aortic lymphadenectomy. In our cohort, the first patient initially underwent sclerotherapy using various agents without effect. Therefore, we tried sclerotherapy using OK-432. This study achieved a 93% clinical success rate. This result is similar to the success rate of sclerotherapy with ethanol or povidone-iodine reported previously (Table 4). However, the mean drainage duration of OK-432 sclerotherapy (7.1 days) was trend to be shorter than that of ethanol and povidone-iodine sclerotherapy (Table 4). Sclerotherapy with ethanol or povidone-iodine was performed in multiple sessions (Sawhney et al., 1996; Zuckerman & Yeager, 1997; Montalvo et al., 1996; Rivera et al., 1996), whereas sclerotherapy with OK-432 in this study was often performed in one session (81%, 13/16), which may have resulted in a shorter drainage period. Because OK-432 is a strong irritant, this study suggests that sclerotherapy with OK-432 can be completed in one session. Three patients who underwent two sessions of sclerotherapy were not infected and the drainage tube output tended to be high volume the day before sclerotherapy (Table 3). Inflammatory change in an infected lymphocele render the lymphocele adhesive (Kim et al., 1999), so sclerotherapy for an infected lymphocele can be performed in one session, but a non-infected lymphocele can be required more sclerotherapy sessions than an infected lymphocele. Also, OK-432 dilution due to high volume of drainage tube output may causes effective to weak. In this study, a major complication (small bowel fistula) was observed in one patient. The patient had an infected lymphocele and was treated with sclerotherapy using OK-432, which resulted in a small bowel fistula. To the best of the authors’ knowledge, there have been no reports of scarring or dysfunction of the surrounding tissues due to OK-432, and the side effects were limited to fever after injection. The small intestine wall was likely fragile due to the infection, and the administration of OK-432 likely caused the fistula. Immediately before administering OK-432, the location of adjacent organs in the lymphocele may have changed from that before percutaneous drainage, and attention should be given to that immediately before administering OK-432. There is no significant correlation between the initial lymphocele size and drainage duration after sclerotherapy (*p* = 0.113). This result is supported by a report by Alago et al. (Alago et al., 2013). Although OK-432 is more expensive than ethanol and povidone-iodine, OK-432 sclerotherapy facilitates early discharge owing to a shorter drainage period, which may reduce the overall hospital costs.

**Table 4:** Studies of various sclerotherapies

References	Agent	Number of lymphoceles	Mean drainage duration after sclerotherapy, days	Success rate, %
Akhan et al. (Akhan et al., 2007)	ethanol	50	11.8	91
Sawhney et al. (Sawhney et al., 1996)	ethanol	14	36	93
Zuckerman and Yeager. (Zuckerman & Yeager, 1997)	ethanol	32	19	94
Alago et al. (Alago et al., 2013)	povidone-iodine	18	13	100
Montalvo et al. (Montalvo et al., 1996)	povidone-iodine	17	36	82
Rivera et al. (Rivera et al., 1996)	povidone-iodine	19	13	62.5
Our study	OK-432	16	7.1	93

This study has some limitations. First, this study had a small sample size and was a retrospective study conducted at a single institution. Further investigation, including treatment results in a large prospective study and comparison of results with other sclerosing agents, is required. Second, the procedure was not standardized owing to variability in the technique according to individual operator preference. However, the drainage period of the OK-432 sclerotherapy was shorter than that of ethanol or povidone-iodine sclerotherapy. Therefore, the results of this study demonstrate the value of this method. It may be necessary to evaluate whether OK-432 is valuable by using an appropriate protocol for the optimal dose and timing of administration.

## Conclusion

OK-432 sclerotherapy is an effective therapeutic method for lymphoceles. It may be possible to shorten the duration of treatment. A major complication was observed in one patient in this study, and attention to adjacent organs may be necessary before administering OK-432.

## Data Availability

All data gathered or analyzed in this study are included in this article.

## Abbreviations

CT: Computed tomography
KE: Klinische Einheit
